# In which clinical scenario would awake fibreoptic nasal intubation be employed?

**DOI:** 10.1002/ccr3.37

**Published:** 2014-01-13

**Authors:** Karim Kassam, Tasmin Rope

**Affiliations:** Northwick Park HospitalLondon, HA1 3UJ, United Kingdom

**Keywords:** Difficult airway, fibreoptic, ludwigs angina

## Abstract

**Key Clinical Message:**

The routine way to access the uncomplicated airway is via direct laryngoscopy. When this is not possible, there are a number of other techniques to help visualization such as the video laryngoscopy. These require a degree of mouth opening. With almost complete trismus, the clinician should resort to awake fibreoptic nasal intubation to secure the airway.

An awake technique is chosen when it is considered unsafe to anesthetize the patient before guaranteeing the ability to secure their airway, usually when difficult laryngoscopy and difficult bag-mask ventilation are expected. This was performed via the nasal route on a 19-year-old man with 10 mm mouth opening with Ludwig's Angina. Ludwig's angina is a rapidly progressing, potentially fulminant cellulitis involving the sublingual, submental, submandibular, and parapharyngeal spaces. Note the supra and subglottic secretions and edema and the swollen arytenoids and vocal cords caused by the infection.

## Supporting Information

**Video 1.** The video of this process is found in the online version of this article.

